# Ungulate responses and habituation to unmanned aerial vehicles in Africa’s savanna

**DOI:** 10.1371/journal.pone.0288975

**Published:** 2023-07-25

**Authors:** Marlice vanVuuren, Rudie vanVuuren, Larry M. Silverberg, Joe Manning, Krishna Pacifici, Werner Dorgeloh, Jennifer Campbell

**Affiliations:** 1 N/a’ an ku sê Foundation, Windhoek, Namibia; 2 Mechanical & Aerospace Engineering, North Carolina State University, Raleigh, North Carolina; 3 Center of Geospatial Analytics, Forestry and Environmental Resources, North Carolina State University, Raleigh, North Carolina; 4 Forestry and Environmental Resources, North Carolina State University, Raleigh, North Carolina; 5 Biology, North Carolina State University, Raleigh, North Carolina; Zonguldak Bülent Ecevit University: Zonguldak Bulent Ecevit Universitesi, TURKEY

## Abstract

This article tests the hypothesis that “the likelihood that the species will react and level at which they do to the unmanned aerial vehicle (UAV) is related to the altitude, number of passes, sound intensity, type of UAV, takeoff distance, and species.” This paper examined the behavioral responses of a group of free ranging ungulate species (Oryx, Kudu, Springbok, Giraffe, Eland, Hartebeest, and Impala) found in an animal reserve in Namibia to the presence of different in-flight UAV models. The study included 397 passes (trials) over 99 flights at altitudes ranging from 15 to 55 meters in three categories of response level: No response, Alert, and Movement. The ungulates were unhabituated to the UAVs and the study was conducted in the presence of stress-inducing events that occur naturally in the environment. Certain species were found to be more reactive than others, in addition to several displaying different response levels in single or mixed herd environments. Zebras were found to be less responsive in mixed herd environments while Oryx were present, as compared to when the Oryx were not; suggesting that some species may respond based on other species perception of threat or their relative fitness levels. The UAVs also produced inconsistent response rates between movement and alert behavior. The reference vehicle, Phantom 3 was much more likely than the Mavic to induce an alert response, while both having similar probabilities of inducing a movement response. Furthermore, the Custom X8 showed significantly more alert and movement responses than the other UAVs. This shows there may be several aspects to the UAVs that affect the responses of the ungulates. For instance, the sound intensity may alert the species more often, but close proximity may induce a movement response. More generally, the data shows that when the UAV is flying above 50 meters and has a measured sound intensity below 50 dB, the likelihood of inducing a movement response on an ungulate species is below 6% regardless of the vehicle on the first pass over the animals. Additionally, with each subsequent pass the likelihood of response dropped by approximately 20 percent. The results suggest a stronger correlation between flight altitude and response across the different ungulates, and the evidence suggests rapid habituation to the UAVs.

## Introduction

The highly valued ungulates in Africa’s savanna, making up more than 25% of its landscape, might be effectively monitored by unmanned aerial vehicles (UAV) [1]. The question of this paper concerns the extent to which ungulate responses might allow aerial wildlife monitoring (AWM) by UAVs. The hypothesis is that ungulate-UAV interaction depends strongly on flight altitude, that flying too low could excessively disturb them, and that there may be a lowest altitude range for which the ungulates are not exceedingly disturbed (putting aside for the moment how exceedingly disturbed is quantified)–dictating some achievable level of discernibility in flight observation. This question is valuable because it strongly influences the future viability of the UAV in the study and protection of the ungulates in Africa’s savanna.

Several papers have already examined the effect of UAV altitude on non-ungulate response (data containing 5 to 100 trials). Biologists found that bears have an increased heart rate when the UAV flies 200 meters from the bear, 20 meters above it [2]. Scientists surveyed elephants in Burkina Faso, flying at altitudes of 100 meters and 300 meters over 10 km transects [3]. Researchers found that the influence of the UAV on the penguin is significant at an altitude of twenty meters [4].

Note that the AWM engineer or designer, when laying out a system of UAVs to monitor a field, would begin by selecting a flight altitude after which vehicles and a communication network would be selected, and in more advanced systems sophisticated data patching methods and graphical interfaces employed [1]. Other preparations, too, would be necessary depending on the application. Indeed, the large number of reported studies and methods preparing for AWM strongly suggests that the potential AWM applications are plentiful. Considering just a few of the papers, studies have focused on the cost savings [5], the management role [1], and more applied work on wildlife tracking [6], counting methods [7–9], and anti-poaching [10].

In general, the level of danger that an ungulate perceives strongly influences its response. The perceived danger that a UAV poses could come from the ungulate-UAV distance, sensed by an auditory signal, or it could come from secondarily observing the responses of neighboring species who have already responded to the UAV. In Africa’s savanna, one finds many ungulates in mixed herds so one could expect neighboring herds to trigger a response. The usual source of the danger to the ungulate comes from the ground, not the air. Therefore, the level of response could be moderate or low depending on flight altitude, while differing from species to species. One also expects the responses to depend strongly on the environment. The time of day (before or after eating), the proximity of nearby animals posing a danger, and the passing by of vehicles or other stress inducing events would be expected to influence the responses.

This paper outlines one of the first longitudinal studies of animal response by a UAV. These operations were conducted in a harsh environment, under high altitude and high heat conditions, where maintaining regular operations was difficult. Locating animals to perform flights was difficult and time consuming and often required considerable hiking for even a limited number of passes and in some circumstances no passes due to failed preflight checks after problems in the field. Even under these unfavorable conditions, the team was able to persevere and acquire 397 passes (trials) over 99 flights.

In this article we examine *how* these ungulates located in the Savannah respond to UAV presence. Specifically, we determine how representing the effects of UAV presence with independent variables that are either continuous, ordinal categorical or qualitative nominal classes might impact our understanding of what drives species reaction. We hypothesized that: (i) altitude (or proximity) is a driving factor and that (ii) the animals will undergo habituation because these ungulates are not normally predated by an aerial foe. To test our hypotheses, we focus on monitoring the wildlife under a variety of conditions in their natural environment through a large data set to see if these factors emerge.

The method section describes the study site, the wildlife species monitored, the data collection process, the aerial vehicles, and the data collected. The results section gives the ungulate responses, the occurrences of responses and discusses the data and secondary factors. Finally, the paper summarizes the results and draws conclusions.

## Materials and methods

### Study area

**Fig 1:**
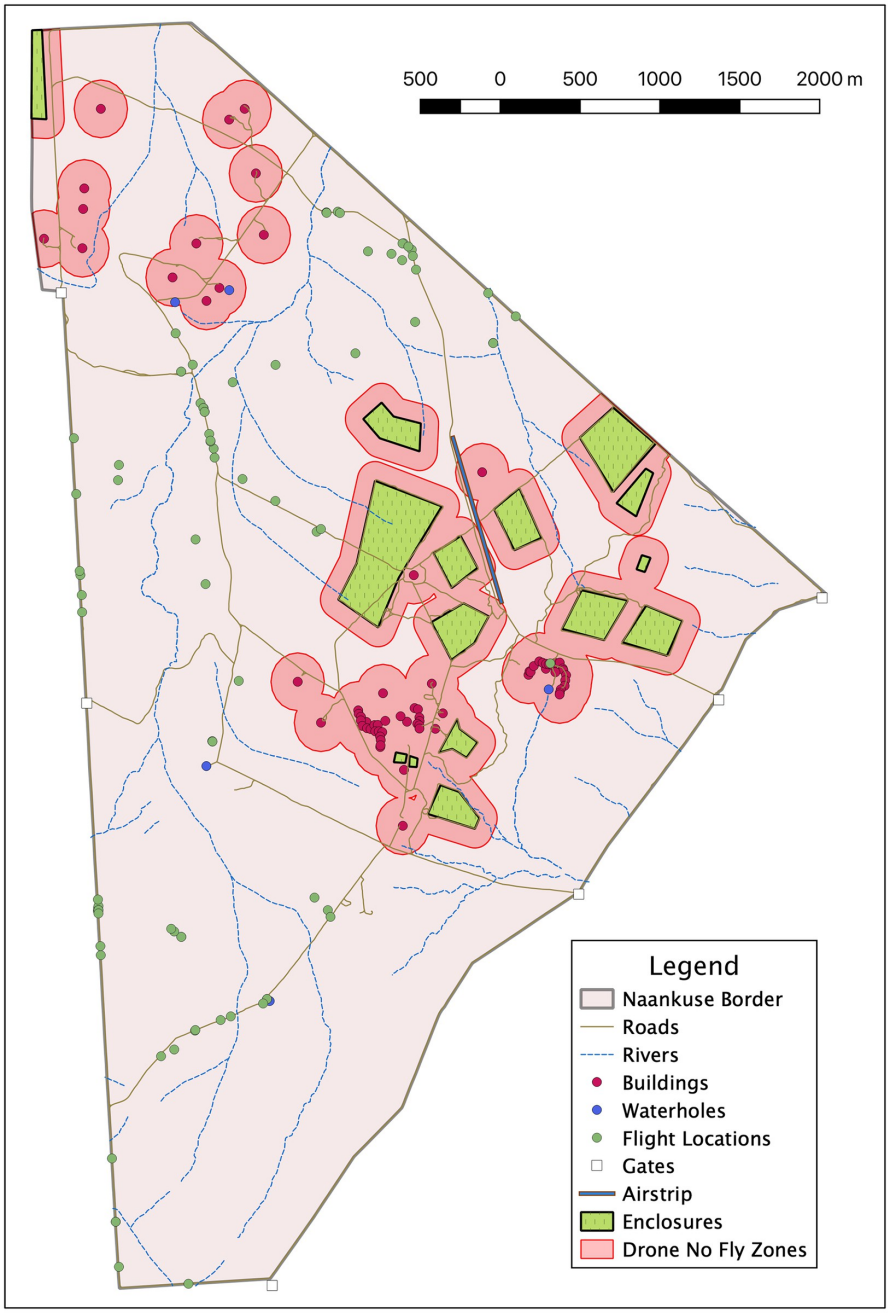
N/a’an ku sȇ map and flight locations. (This figure was created under CC BY 4.0).

The study was conducted at a wildlife sanctuary (N/a’an ku se) located about 40 km east of Windhoek, Namibia’s capital city, over a ten-week period in 2017 from September to November. Permits to operate UAVs of this size were not necessary at the time of flight within the country. The landowners are coauthors on the article and helped identify the need to understand UAV and animal interaction for safe monitoring of wildlife for future use of UAVs in Africa’s savannah. North Carolina State Universities Animal Care and Use Committee (IACUC) was contacted to ensure the welfare of the animals was not governed by any rules or regulations given the nature of the study protocol. They determined that no IACUC review was required for this study. The land is 25 km^2^, located on Namibia’s central plateau, and its elevation is 1600 to 1800 meters. From September to November, the average temperature is 20 to 23 C, and the average rainfall is 10 mm in September, 10 mm in October, and 30 mm in November. The land is a semi-closed eco-system surrounded by an 8-foot game fence that includes free ranging leopards and cheetahs and the species are unhabituated to UAVs. Fig 1 is a map of the test site created by gathering all the relevant GPS coordinates (i.e., property boundaries, enclosures, building corners, etc.) by walking the property and then using QGIS to create a map [11]. The study species included a group of ungulates commonly found in arid savannah eco-systems throughout Africa: Oryx (*Oryx gazella*), Eland (*Taurotragus oryx*), Giraffe (*camelopardalis*), Springbok (*Antidorcas marsupialis*), Hartebeest (*Alcelaphus buselaphus*), Plains Zebra (*Equus quagga*), and Kudu (*Tragelaphus strepsiceros*).

A field unit of 12 students collected data over a 10-week period (See the acknowledgement section). The field unit formed data collecting sub-units, each consisted of four personnel: (1) a pilot, (2) a primary observer, (3) a ground camera operator, and (4) a data recorder. The pilot was responsible for flying the UAV. When flying in radio controlled (RC) mode, the pilot was also responsible for operating the flight camera and maintaining visual contact with the study species from the air. The primary observer was responsible for observing the species and maintaining visual contact with the ground station. The primary observer worked next to the data recorder and relayed all of their wildlife response observations throughout the flight to the recorder. The ground camera operator was responsible for photographing/video recording the responses. The data recorder was responsible for annotating information relayed from the primary animal observer on a flight-data collection sheet.

### Experimental design

The purpose of this study was to assess ungulate responses to UAVs flying at different altitudes in Africa’s arid savannah. Several considerations were vital when setting up the study. First, to ensure minimally impacted studies from human interaction, the study should be conducted in a manner as unobtrusive to the ungulate wildlife such that little or no levels of habituation are required. Furthermore, when none of the conditioning is negative, habituation would only tend to decrease the altitude that an ungulate tolerates. Therefore, for the purposes of this study, it was reasonable to focus on unhabituated ungulates. Secondly, in order for the results to apply to the natural habitat of the savannah, it was also important to perform the study in the mixed herd environment in the presence of its naturally occurring stress inducing events. The variable conditions of the natural environment introduce noise into the study which increases the difficulty to discern the effect of altitude on the response data. However, it can also help determine whether or not UAV altitude is a dominant factor against the other factors, as hypothesized.

The ungulate response was gathered at four levels. The levels were designated by the following: No Response (no discernable movement), Alert (eye/head movement and/or turning of the upper body), Relocation (moving out of the way of the UAV but still in sight), and Flee (significant movement from UAV location). Due to the very low number of flee responses and the similarity of the movement aspect, the two location movement responses (Relocation and Flee) were combined into one category (Movement), which was categorized as relocation away from the UAV. Therefore, the data were analyzed by three categories (No Response, Alert, Movement).

The suite of UAVs consisted of the DJI Phantom 3 (the reference vehicle), DJI Mavic Pro, Custom X8 (Also referred to as an Octocopter), and Sky Eye. Table 1 shows the tested noise levels of the different UAVs with their baseline equivalent altitudes.

**Table 1:** UAV noise levels.

UAV type	Frame size (mm)	Weight (g)	Noise level (dBA)	Altitude (m)
Phantom 3	350	1280	60.24	50
	Reference altitude			50
Mavic Pro	335	734	45.7	50
	Phantom equivalent altitude			266.7
Sky Eye	2000	2000	52.5	50
	Phantom equivalent altitude			121.9
Custom X8	650	4400	65.0	50
	Phantom equivalent altitude			28.9

As shown, the loudest to quietest vehicles were the Custom X8, Phantom 3, Skyeye and Mavic Pro. Noise disturbance is not only influenced by the noise which can be qualified by sound intensity (dB) and frequency (Hz), but also by its duration and pattern. The age and physiological state of the animal at the time of exposure, the exposure history of the animal, and the predictability of the acoustic stimulus can also play a role [12]. The effect of altitude on animal response was studied in the presence of all of these naturally occurring factors.

The design of the experiment began with determining a range of flight altitudes. The range of flight altitudes were determined through 23 preliminary flights, flying at altitudes from 20 meters to 100 meters in 10-meter increments. We determined that 15 meters was a suitable lower bound because of safety concerns and that 55 meters was a suitable upper bound because negative response had already significantly dropped off by that altitude. The data collection process began every morning with locating animal herds and setting up a field unit nearby. Local personnel understood the best locations to set up field units and launch the UAVs. As shown in Fig 1, the launch points were located in the low-lying, open areas along the roads. The data collecting personnel hiked or travelled by ground vehicle to those launch points.

The horizontal distances between the animals and the field unit points varied widely–between 50 and 600 meters (measured in 25 m increments). The total data set consisted of 397 passes (trials) over 99 flights, however, the analyzed dataset not including preliminary flights was 346 passes over 76 flights that ranged in altitude from 15 to 55 meters. Each pass began with taking off from a launch point, flying about 200 m away from a spotted animal group, changing altitude as appropriate, and then flying 400 m, passing over the animal group. The UAV was flown over the animal or herd at 10 m/s. The next pass was flown at another randomly selected altitude. If a movement response was invoked (relocation or flee), the UAV would wait at a distance while the animals would settle back to a sedentary behavior. Passes were continued until battery levels were too low to continue or the animals were no longer within sight. Table 2 shows the procedure that each of the members of the field unit followed.

**Table 2:** Field unit procedures.

Pilot	Pre-flight checklist, flight, post-flight checklist, reports damage or concerns at end of day
Camera operator	Pre-flight and post-flight checklist for camera and gimbal, ensure the data are downloaded, clear SD at end of day, assist pilot with UAVs.
Animal observer	Observes surrounding field prior to takeoff for safety, observes animal responses during flight, decides whether to continue or end mission based on animal behavior.
General observer	Ensures all of the roles are being properly executed, ensures that no wildlife is encroaching upon during the flights, assists the data collector in obtaining data during the flights when several tasks are being performed simultaneously.
Data entry	Enters all of the pre, during, and post flight data

### Statistical analysis

An ordinal logistical regression was chosen due to the ordered nature of the response between No Response, Alert, and Movement. These models were developed using R 4.0.5 (R Development Core Team 2021). Several model generating techniques were used from three packages, the polr function from the MASS package [13], vglm from the VGAM package [14], and the cumulative link model (clm) from the ordinal package [15]. These different models were useful in testing variable significance, developing the best fit model, testing the proportional odds assumption, and lastly fitting partial proportional odds models for variables that do not conform to the proportional odds assumption.

The first model was started by using the polr function, which comes from proportional odds logistical regression, in the MASS package (the proportional odds assumption will be tested later in this analysis). Considering the large number of variables, the final models’ variables were chosen using the Akaike information criterion (AIC). The AIC is an estimator of the relative quality of a model for a given data set by dealing with the tradeoff between goodness of fit and simplicity. The variables were first tested for independence before entering the model using the chi-squared test for independence. The best model was chosen by minimizing AIC to an extent and in close situations allowing the discretion of the researchers. Several iterations using the step function were run in forward, backward, and both direction configuration to confirm the best fitting model. An initial model containing all variables was fitted and run with an ANOVA type II test for significance of variables. This all-variable model was compared to the best fitting model to evaluate variable contributions. The variables that were left out were found to not have meaningful influence or significance. The final model was then chosen after analyzing that there were sufficient data to create a good fitting model given the current data set. Furthermore, the model was then tested to meet the proportional odds assumption and ensured that the model was an accurate representation of the data through various methods detailed in the results.

## Results

### Data

The data set used in analysis did not include preliminary flights for testing and was comprised of 346 passes over 76 flights. The data consisted of species reactions (No Response, Alert, and Movement). Of these reactions, 164 (47.4%) were No Response, 115 (33.2%) were Alert, and 67 (19.4%) were Movement. The research hypothesis posed to the data was that “the likelihood that the species will react and level at which they do to the UAV is related to the altitude, number of passes, sound intensity, type of UAV, takeoff distance, and species.” Thus, the outcome variable, Response, was the herds reaction to the UAV after a pass taken overhead. The predictors were Altitude (a continuous variable using 15, 25, 35, 45, and 55 meters), Pass (the pass number over the herd during a given flight), Drone (Mavic, Phantom 3, Custom X8, Sky Eye), Sound Intensity (dB level that would have been experienced by animal herd estimated using altitude at pass an noise characteristic of vehicle), takeoff distance (herd distance from ground crew at takeoff measuring in meters), as well as each animal species with sufficient samples which were Oryx, Giraffe, Springbok, and Zebra in both single species and mixed group.

### Species reactions

The relative abundance of the different species varies from site to site and by season. Table 3 shows the populations of the monitored species at the test site. In some cases, single species herds were observed while in other cases there were mixed herds.

**Table 3:** Number of passes (% of total passes).

Species	Single Species	Mixed Herd
Oryx	50 (14.45%)	120 (34.68%)
Giraffe	46 (13.29%)	14 (4.05%)
Springbok	46 (13.29%)	58 (16.76%)
Zebra	27 (7.80%)	87 (25.14%)
Other	39 (11.27%)	49 (14.16%)

As previously stated, the data were broken up by distinguishing between a single species encounter and a mixed herd encounter. Eland, Hartebeest, Impala, and Waterbuck were also found on the property; however, they were not passes over in enough flights to include in the species analysis. Therefore, the species predictors used were Oryx Single, Zebra Single, Springbok Single, Giraffe Single, Oryx Mixed, Zebra Mixed, Springbok Mixed, and Giraffe Mixed.

### Ordinal regression analysis

A 13-predictor ordinal logistical regression was fitted to the data to test the hypothesis relationship regarding the likelihood that a species will react based on the flight altitude, number of passes, sound intensity, type of UAV, takeoff distance, and species. The Akaike Information Criterion (AIC) was used to find the best fitting model. This was done by eliminating predictors to minimize the models AIC score. This process was done in a backwards (starting with all of the predictors and eliminating), forward (Starting with no predictors and adding), and both directions (starting with a number of predictors and adding and eliminating based on AIC score) to find the best fitting model. Two models were found with similar AIC, both with the same predictors except one contained altitude and the other dB. Interestingly, the sound intensity predictor was calculated from the altitude but did not show any correlation when tested. This was likely due to the large difference in the level of sound produced by the different UAVs.

The parallel lines test was done to test proportional odds assumption by comparing it to a general model (a saturated multinominal logistical regression). The comparison was done between the general model and using the Chi-Squared test to the multinomial logistical regression using the multinom() function from the nnet package [13]. The test showed the general model gives a significantly better fit (p<0.05) thereby inferring that the proportional odds assumption was not valid. However, the test of the proportional odds assumption has been described as anti-conservative, that it nearly always results in the rejection of the proportional odds assumption particularly when the number of explanatory variables is large [16], the sample size is large, or there is a continuous explanatory variable [17]. The model was developed to counter this and was built in the ordinal package with the clm() function and tested the significance of the proportional odds assumption in using the nominal() function. The variables Drone and ZebraMixed were significant in the test indicating that the proportional odds assumption was broken. The Odds Ratios were viewed in the different model configurations to see if the estimators truly were different, and it was deemed that both Drone and ZebraMixed did not meet the proportional odds assumption.

The models were then developed as partial proportional odds models relaxing the proportional odds assumption for ZebraMixed and Drone. Finally, the model was tested again against the more general model and the general model was not found to be a significantly (p<0.05) better fit. The following table is the summary of the model. Evaluation of the models were done by taking an overall model evaluation, statistical tests of the individual predictors, goodness of fit statistics, and validation of predicted probabilities.

### Models without interaction effects

The first models developed were those without interaction effects. The two best fitting models are listed in Table 4 with the odds ratios and confidence intervals. The factors that did not influence the model significantly are takeoff distance, Oryx Single, Zebra Single, Springbok Single, Oryx Mixed, Springbok Mixed, and Giraffe Mixed.

**Table 4:** Model 1 and 2 summaries.

*Predictors*	Model 1			Model 2		
	*Odds Ratios*	*CI*	*p*	*Odds Ratio*	*CI*	*p*
(Intercept) * 1	17.71	7.56–41.52	**<0.001**	0.00	0.00–0.00	**<0.001**
(Intercept) * 2	1.46	0.64–3.32	0.372	0.00	0.00–0.00	**<0.001**
Altitude	0.94	0.92–0.96	**<0.001**			
Pass	0.81	0.72–0.90	**<0.001**	0.80	0.72–0.90	**<0.001**
Drone [Mavic] * 1	0.38	0.22–0.69	**0.001**	9.01	3.33–24.38	**<0.001**
Drone [Mavic] * 2	1.02	0.50–2.06	0.960	24.12	7.84–74.21	**<0.001**
Drone [Sky Eye] * 1	0.66	0.25–1.75	0.450	3.77	1.30–10.89	**0.014**
Drone [Sky Eye] * 2	1.15	0.33–4.04	0.822	6.69	1.73–25.87	**0.006**
Drone [Octo Copter] * 1	8.83	2.82–27.65	**<0.001**	3.13	0.99–9.88	0.051
Drone [Octo Copter] * 2	7.09	2.88–17.45	**<0.001**	2.61	1.06–6.45	**0.038**
GiraffeS [TRUE]	3.05	1.58–5.87	**0.001**	3.02	1.57–5.81	**0.001**
ZebraM [TRUE] * 1	2.07	1.12–3.80	**0.020**	2.02	1.10–3.70	**0.024**
ZebraM {TRUE] * 2	4.60	2.26–9.36	**<0.001**	4.46	2.18–9.12	**<0.001**
dB				1.24	1.17–1.32	**<0.001**
Observations	346			346		

**Model 1**: Response ~ Altitude + Pass + Drone + GiraffeSingle + ZebraMixed

**Model 2**: Response ~ dB + Pass + Drone + GiraffeSingle + ZebraMixed

#### Overall model evaluation

The overall model evaluation was done by comparing the model to an intercept only model (called the null model). Consequently, the significant chi-squared statistic (LR stat. = 102.23, Pr(Chi) = 0) for model 1 and (LR stat. = 103.18, Pr(Chi) = 0) for model 2 indicates the models containing predictors give a significant improvement over the baseline intercept-only model.

#### Statistical tests of individual predictors

The statistical significance of individual regression coefficients (i.e., betas) is tested using the Wald chi-squared statistic (Table 5). As seen in Table 5, Pass, Drone, Giraffe Single, and Zebra Mixed were significant predictors in both models as well as altitude in model 1 and dB in model 2 in the response to UAVs flying overhead of the herds (p<0.05) and even accounting for the Bonferroni correction of Drone (p<0.0083) and ZebraMixed (p<0.025).

**Table 5:** Anova type II evaluation of model.

Model 1				Model 2			
	Df	Chisq	Pr(>Chisq)		Df	Chisq	Pr(>Chisq)
Altitude	1	49.38771	0.000	dB	1	51.14279	0.000
Pass	1	14.52715	0.000	Pass	1	15.34127	0.000
Drone	6	41.10814	0.000	Drone	6	38.67013	0.000
GiraffeSingle	1	11.08581	0.000	GiraffeSingle	1	10.98098	0.001
ZebraMixed	2	17.75902	0.000	ZebraMixed	2	16.84586	0.000

#### Goodness-of-fit statistics

Goodness-of-fit statistics assess the fit of logistical model against actual outcomes. Three inferential goodness-of-fit tests were performed on the model: the Lipsitz goodness of fit test, the Pulkstenis-Robinson chi-squared test, and Pulkstenis-Robinson deviance test. Table 6 below shows the results of the tests. The Lipsitz test yielded a (model 1 = X^2^ (df = 9) of 7.6884, model2 = X^2^ (df = 9) of 11.965) and was insignificant (p>0.05), suggesting the models fit well to the data confirming the null hypothesis. Similar findings were concluded with the PR Chi-Squared test and the PR deviance test both yielding significant values (p>0.05) and confirming the null hypothesis that the model is a good fit to the data. Using both the PR test and Lipsitz test helps fully grasp the goodness of fit as PR tests work best when the lack of fit is associated with categorical variables, whereas the Lipsitz test works best when continuous covariates drive the lack of fit [18, 19].

**Table 6:** Goodness-of-fit tests.

	Model 1			Model 2		
	X^2^	df	p-value	X^2^	df	p-value
Lipsitz	7.6884	9	0.566	11.965	9	0.215
Pulkstenis-Robinson X^2^	6.401	12	0.895	6.548	12	0.891
Pulkstenis-Robinson deviance	7.256	12	0.840	7.416	12	0.829

#### Validation of predicted probabilities

The resultant predicted probabilities were then compared with the actual outcome to determine if high probabilities are indeed associated with events and low probabilities with non-events. The degree to which predicted probabilities agree with actual outcomes is expressed in the Table 7, the classification table. The overall correction prediction was 60.1% for model 1 and 59.8% for model 2, a sizable improvement over the chance level (33.3%). In the opinion of Hosmer and Lemeshow (2000, p. 160), “the classification table is most appropriate when classification is a stated goal of the analysis; otherwise, it should only supplement more rigorous methods of assessment of fit.” It was determined that based on the classification table and the goodness-of fit models that the data were properly fit to the model.

**Table 7:** Classification table.

Observed	Model 1 Predicted				Model 2 Predicted			
	No Response	Alert	Movement	% Correct	No Response	Alert	Movement	% Correct
No Response	122	33	9	74.4	125	31	8	76.2
Alert	44	64	7	55.7	48	59	8	51.3
Movement	19	23	25	37.3	22	22	23	34.3
% Correct	65.9	53.3	61.0	60.1	64.1	52.7	59.0	59.8

Interaction effects were added based on the underlying main effects. For both models, the interaction effects between Altitude or dB (model 1 or model 2 respectively), Pass, and Drone were tested as well as the ZebraMixed with other mixed group species. Based on this analysis significant interaction effects were found in model 1 between Altitude and Pass as well as ZebraMixed and OryxMixed and similarly with model 2 between dB and Pass and ZebraMixed and OryxMixed. There was significance found between Drone and (Altitude and dB) but given the low number of samples with the Sky Eye and Custom X8, the model seemed to be saturated with some of the limited amounts of data. Similar to the initial model evaluation, the models containing interaction effects had an overall evaluation, statistical tests for individual predictors, goodness-of-fit tests, and validation of the predicted probabilities.

### Models containing interaction effects

The second set of analyzed were those with interaction effects. The two best fitting models are listed in Table 8 with the odds ratios and confidence intervals. Similar to the models without interaction effects, the factors that did not influence the model significantly are takeoff distance, Oryx Single, Zebra Single, Springbok Single, Springbok Mixed, and Giraffe Mixed.

**Table 8:** Model 3 and 4 summaries.

*Predictors*	Model 3			Model 4		
	*Odds Ratios*	*CI*	*p*	*Odds Ratio*	*CI*	*p*
(Intercept) * 1	3.72	1.10–12.54	**0.034**	0.00	0.00–0.00	**<0.001**
(Intercept) * 2	0.28	0.08–0.98	**0.046**	0.00	0.00–0.00	**<0.001**
Altitude	0.98	0.95–1.02	0.330			
Pass	1.24	0.94–1.65	0.130	0.29	0.12–0.73	**0.008**
Drone [Mavic] * 1	0.33	0.18–0.62	**<0.001**	8.94	3.22–24.85	**<0.001**
Drone [Mavic] * 2	0.98	0.48–2.01	0.954	25.95	8.18–82.33	**<0.001**
Drone [Sky Eye] * 1	0.60	0.22–1.66	0.325	3.49	1.14–10.64	**0.028**
Drone [Sky Eye] * 2	1.15	0.32–4.22	0.83	6.52	1.61–26.40	**0.009**
Drone [Octo Copter] * 1	9.91	3.07–32.02	**<0.001**	3.68	1.15–11.81	**0.028**
Drone [Octo Copter] * 2	8.28	3.28–20.94	**<0.001**	3.23	1.27–8.23	**0.014**
GiraffeS [TRUE]	3.62	1.80–7.27	**<0.001**	3.58	1.80–7.11	**<0.001**
ZebraM [TRUE] * 1	11.72	3.13–43.88	**<0.001**	11.42	3.07–42.46	**<0.001**
ZebraM {TRUE] * 2	24.03	6.39–90.29	**<0.001**	23.18	6.22–86.39	**<0.001**
OryxM [TRUE]	1.68	0.79–3.58	**0.177**	1.77	0.83–3.75	**0.137**
ZebraM {TRUE] * OryxM [TRUE]	0.10	0.02–0.44	**0.002**	0.10	0.02–0.45	**0.003**
Altitude * Pass	0.99	0.98–0.99	**0.001**			
dB				1.19	1.10–1.28	**<0.001**
dB * Pass				1.02	1.00–1.03	**0.029**
Observations	346			346		

**Model 3**: Response ~ Altitude + Pass + Drone + GiraffeSingle + ZebraM*OryxM + Altitude:Pass

**Model 4**: Response ~ dB + Pass + Drone + GiraffeSingle + ZebraM*OryxM + dB:Pass

#### Overall model evaluation

The overall model evaluation was done by comparing the model to an intercept only model (called the null model). Consequently, the significant chi-squared statistic (LR stat. = 122.28, Pr(Chi) = 0) for model 3 and (LR stat. = 118.13, Pr(Chi) = 0) for model 4 indicates the models containing predictors give a significant improvement over the baseline intercept-only model. This was expected since the models were only adding interaction effects.

#### Statistical tests of individual predictors

The statistical significance of individual regression coefficients (i.e., betas) is tested using the Wald chi-squared statistic (Table 9). As seen in Table 9, all the predictors in Model 3 showed significance except for OryxMixed. Conversely, in Model 4 the interaction effect is not considered significant when using the Holm-Bonferroni correction. It is the second largest effects so would need to be p< 0.025, therefore, any p-value larger (OryxMixed) is also not significant.

**Table 9:** Anova type II evaluation of model.

Model 3				Model 4			
	Df	Chisq	Pr(>Chisq)		Df	Chisq	Pr(>Chisq)
Altitude	1	42.903	0.000	dB	1	49.156	0.000
Pass	1	11.818	0.001	Pass	1	14.337	0.000
Drone	6	42.595	0.000	Drone	6	40.875	0.000
GiraffeSingle	1	13.065	0.000	GiraffeSingle	1	13.297	0.000
ZebraMixed	2	14.471	0.001	ZebraMixed	2	14.295	0.001
OryxMixed	1	0.025	0.873	OryxMixed	1	0.000	0.994
ZebraM:OryxM	1	9.216	0.002	ZebraM:OryxM	1	9.074	0.003
Altitude:Pass	1	10.279	0.001	dB:Pass	1	4.766	0.029

#### Goodness-of-fit statistics

Goodness-of-fit statistics assess the fit of logistical model against actual outcomes. Three inferential goodness-of-fit tests were performed on the model: the Lipsitz goodness of fit test, the Pulkstenis-Robinson chi-squared test, and Pulkstenis-Robinson deviance test. Table 10 shows the results of the tests. The Lipsitz test yielded a (model 1 = X^2^ (df = 9) of 10.852, model2 = X^2^ (df = 9) of 12.442) and was insignificant (p>0.05), suggesting the models fit well to the data confirming the null hypothesis. Similar findings were concluded with the PR Chi-Squared test and the PR deviance test both yielding significant values (p>0.05) and confirming the null hypothesis that the model is a good fit to the data.

**Table 10:** Goodness-of-fit tests.

	Model 3			Model 4		
	X^2^	df	p-value	X2	df	p-value
Lipsitz	10.852	9	0.286	12.442	9	0.190
Pulkstenis-Robinson X^2^	12.904	12	0.376	7.777	12	0.802
Pulkstenis-Robinson deviance	16.012	12	0.191	10.035	12	0.613

#### Validation of predicted probabilities

Table 11 presents the predicted probabilities. The resultant predicted probabilities were then compared with the actual outcome to determine if high probabilities are indeed associated with events and low probabilities with non-events. The degree to which predicted probabilities agree with actual outcomes is expressed in the classification table (Table 11). The overall correction prediction was 64.6% for model 3 and 61.0% for model 4, a great improvement over the chance level (33.3%).

**Table 11:** Classification table.

Observed	Model 3 Predicted				Model 4 Predicted			
	No Response	Alert	Movement	% Correct	No Response	Alert	Movement	% Correct
No Response	124	34	6	75.6	124	30	10	75.6
Alert	34	72	9	62.6	44	61	10	53.0
Movement	15	24	27	40.9	20	21	26	38.8
% Correct	71.7	55.4	64.3	64.6	66.0	54.5	56.5	61.0

## Discussion

As stated previously, the 4 models were chosen because of their Akaike information criterion (AIC) score relative to other models containing different sets of predictors. Model 1 (619.59) and Model 2 (618.95) had the best AIC scores and were very close in value for the models without interaction effects. Both models were retained for analysis and from which to evaluate the key predictors. It was important to first analyze the predictors without interactions to understand how the predictors alone effected the Response, after which the interaction effects were added and their influence on the models evaluated. When the models were developed with interaction effects the scores were not quite as close and the score for the model containing altitude instead of dB scoring lower (Model 3: 606.59, Model 4: 611.61). Model 3 had the best AIC score and the highest overall prediction at 64.6%. All four models consistently predicted more false positives than false negatives. This was to be expected as there were a lot of other factors in the natural habitat to create response. It was also expected as a species will generally react in most circumstances over not reacting due to the consequences. It is in the animal’s best interest to react to an undetermined threat as the consequence could be grave for not reacting, while the opposite is not true. And on the same note, these models were not meant for predictive purposes, but to understand the influence of UAVs more generally on ungulate species.

### Species

The field unit observed the animal’s response with the effect of their proximity to the UAV, the time of day, and whether the UAV appeared to be competing with attention of a larger source of danger. These other sources include vehicles that drive by, nearby baboon/cheetah walks, and weather, as well as general species behavioral patterns. Generally, most ungulates graze or browse in the early morning and late afternoon/early evening, allowing them to avoid the heat during midday by hiding under the shade of trees. Some ungulates are browsers (eat leaves) and are found in thick bush, making them more difficult to spot and observe. Others are grazers, found in open grassland, and are easier to observe. Impala and Kudu numbers were lower simply because fewer of them are free ranging on the property. Kudus are very cryptic/elusive animals, often hiding in bush cover and in small herds. Most of the data were collected from larger open areas where the animals were easier to locate, and where they could be observed from a distance without disturbing them prior to take-off.

More generally, pertaining to animal habituation to humans and traffic, we used vehicles to transport ourselves and the vehicle traffic appeared to affect the responses. We observed the animals to be flightier in areas that are closer to more traveled roads, and it was difficult to collect data close to neighboring farms that allow game hunting since the animals there were flighty when sensing human presence. Male responses may have also differed from female responses. For example, some male springboks may have suppressed their flight responses to keep hold of their territory while the females and maternity herds exhibited a flight response.

**Fig 2:**
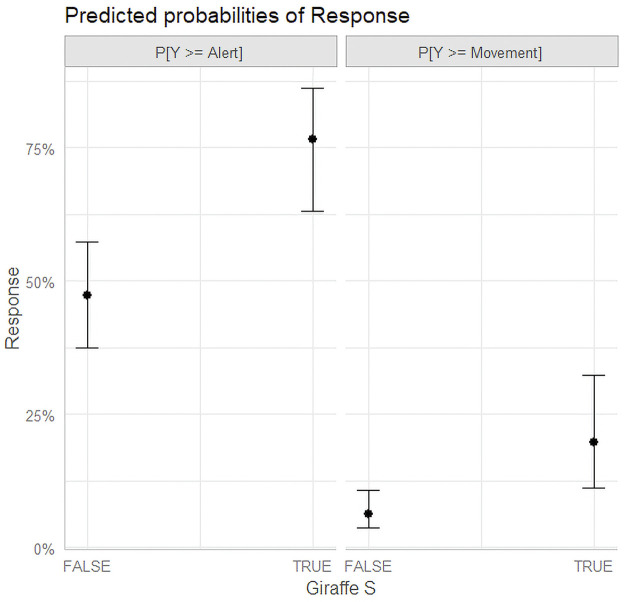
Predicted probabilities of response by Giraffe.

From the models we see that both Giraffe in single species herd were significant as well as Zebra in mixed herd environments. The Giraffe did not violate the proportional odds assumption. Therefore, the odds of inducing a No Response reaction over an Alert reaction is equal to inducing an Alert reaction over a Movement reaction. The given values single species giraffe had an odds ratio of 3.05 with a 95% confidence interval ranging from 1.581 to 5.871 in the no interaction model and 3.62 with a confidence interval ranging from 1.802–7.266 in the model including interaction terms. Both terms do overlap and are found within each other’s confidence intervals. Fig 2 shows the predicted probabilities of response of the Giraffe adjusted for Altitude = 35m, Pass = 3, Drone = Phantom 3, ZebraM & OryxM = False.

**Fig 3:**
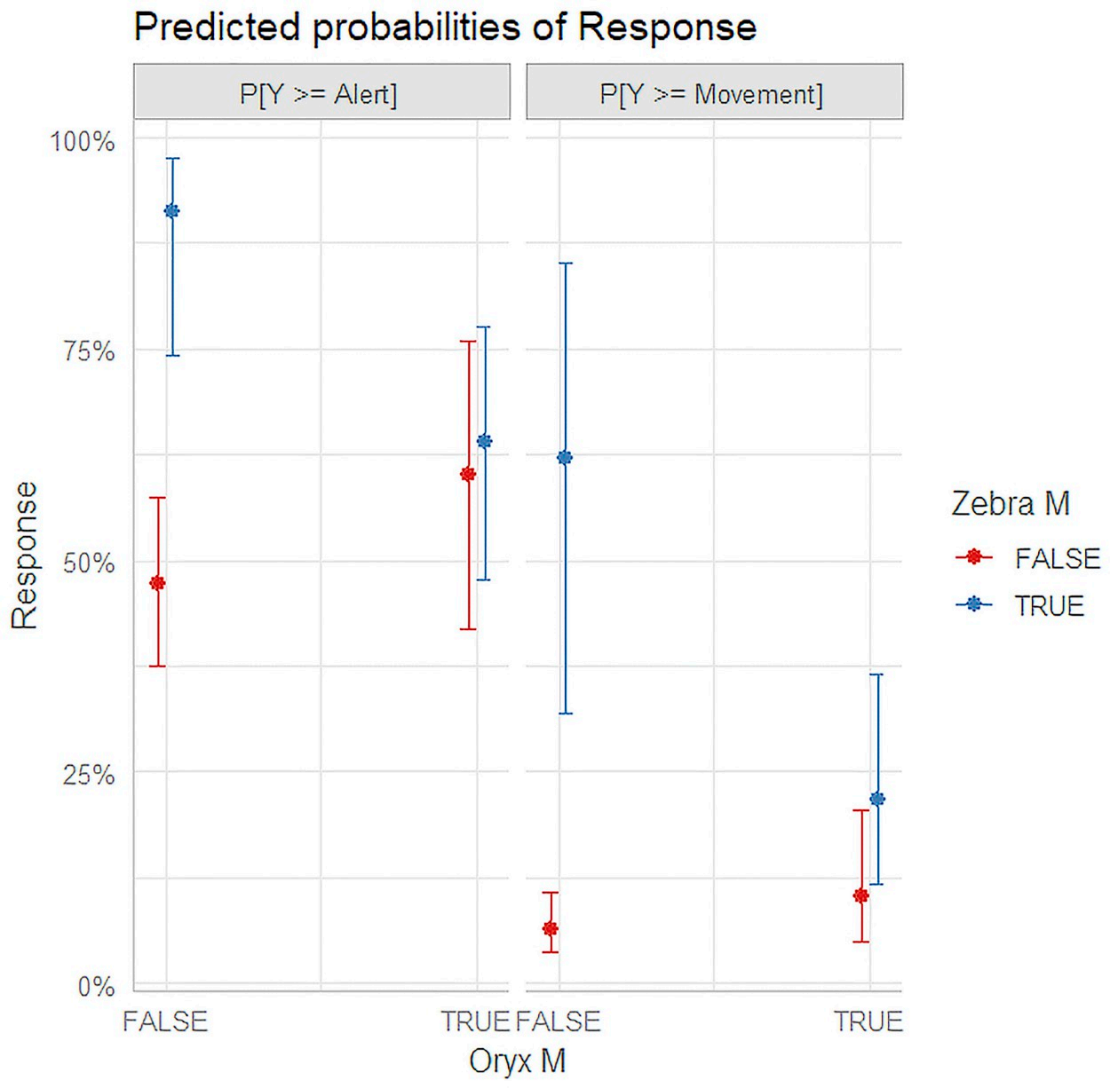
Predicted probabilities of Zebra and Oryx response in mixed herd environments.

More interestingly, the Zebra was found to have a significant effect in mixed herd environments and the interaction term was significant between the Zebra and Oryx in mixed herd environments. Fig 3 shows the predicted probabilities of Zebra and Oryx in mixed herd environments adjusted for Altitude = 35m, Pass = 3, Drone = Phantom 3, Giraffe Single = False.

The response is found to be similar when Oryx is in the mixed herd environment as well as the Zebra. This is not found to be the case when Oryx is not present. The Zebra in the mixed herd environments without Oryx create a much higher probability of response than without them. This indicates that some species do react more than others, just like Giraffe in single species environments, and also implies that some species (Oryx in this case) can mitigate the reaction. While Zebra may normally be a more reactive species, when Oryx is present in the mixed herd, they become less reactive or may look to the Oryx as to their perception of the threat. This may also be attributed to different species comparative fitness levels in the mixed herd.

### UAV

**Fig 4:**
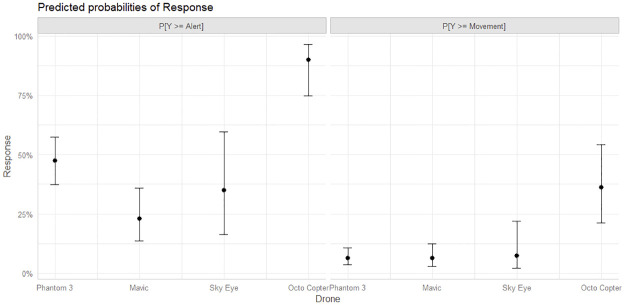
Predicted probabilities based on UAV type.

There were considerable differences within the suite of UAVs from size, speed, sound profile, to mode of flight. Through these differences the predictor was tested to see if any UAV created a higher response compared to other UAVs. The Phantom 3 was considered the reference UAV in most of the previous calculations, but we can see how the species response would vary considerably based on the UAV flown. Fig 4 shows the predicted probability of a greater than or equal response to Alert on the left and Movement on the right for each Drone and is adjusted for and Altitude = 35m, Pass = 3, Giraffe Single = False, and Oryx Mixed & Zebra Mixed = False.

The Custom X8 (Octo Copter) has a significantly higher probability of inducing a response. The proportional odds assumption was not met for the predictor Drone so the odds of inducing a response vary between the two interaction interfaces. The Custom X8 has a 90% predicted chance of inducing an alert response with the 95% confidence interval encompassing up to 96%. That is considerably higher than the other UAVs. Additionally, the UAVs do not have consistent response rates. While the Phantom 3 is much more likely than the Mavic to induce an alert response, they have very similar probabilities of inducing a movement response. This suggests there may be several aspects to the UAVs that affect the response of the ungulates. For instance, the sound intensity may alert the species more often, but close proximity may induce a movement response.

### Altitude and pass

**Fig 5:**
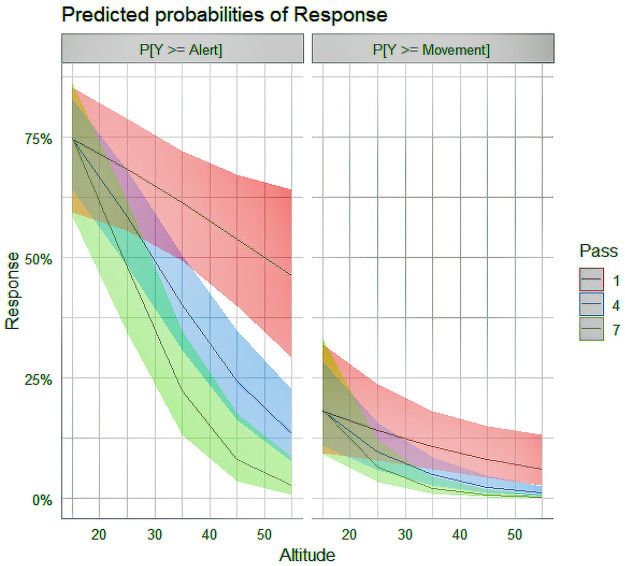
Predicted probabilities based on UAV altitude and pass number.

Pass was considered to understand the reaction based on the UAVs continued presence in the area. The UAV would continue passes until the battery was too low or the herd left visual range. As seen in model 3, there was a significant interaction effect between Altitude and Pass. Fig 5 shows the predicted probabilities based on altitude and pass adjusted for Drone = Phantom 3, Giraffe Single = False, OryxMixed & ZebraMixed = FALSE.

As seen in Fig 5, species response drops significantly based on the number of passes unless at low altitudes. This suggests that the ungulate species undergo rapid habituation to the UAV. This was expected as ungulates do not have any aerial threats. The presence of the UAV likely alarms them, but they very quickly settle once they perceive that the UAV is not a threat. However, once the UAV reaches a certain low level of altitude, the ungulates don’t rapidly habituate, or it may take considerably longer to habituate at such close proximity. This is similar to an ungulate perceived threat from other vehicle types approaching (i.e., cars). Ungulates are more reactive in very remote areas than those in highly trafficked locations.

### Altitude vs Sound intensity

While the sound intensity models fit well with the data, the researchers believe the evidence is not compelling enough to ascribe the animal response to sound intensity over altitude. Sound intensity was added as a predictor because of the much higher responses to the Custom X8, which was a much louder vehicle. This led the researchers to believe that sound intensity could be the contributing factor over altitude alone. Therefore, the sound profiles of the vehicle were measured in an anechoic chamber under similar conditions to attempt to differentiate between the different levels of inducing responses.

Several factors led the researchers to believe that altitude was a better predictor than sound intensity. First, and very apparently, in the model associated with sound intensity, the Beta’s for each individual UAV were different. If sound intensity were the dominating factor, then these would be the same or close. These Beta’s showed significance between the Phantom 3 and Mavic. Similarly, the differences in response levels from the varying UAVs at similar altitudes appear to illuminate multiple factors at play associated with the UAVs. Therefore, altitude as a predictor likely encompasses more of these factors because proximity (intensity) to these varying characteristics likely plays a role as well.

Second, the interaction effect between sound intensity and pass was not significant. If the animal is becoming habituated to the vehicle flying overhead, it would be anticipated that they would also become capable of enduring slightly more of the effector (i.e., sound intensity) and having the same response, like we see with altitude. For instance, if the UAV passes over a second time at the same altitude, the results show it will induce less of a response. Similarly, if passed at a lower altitude on the second pass, it would induce the same probability as the first pass, which we see in the data for altitude. This same interaction would be expected from sound intensity. If sound intensity where the leading factor over altitude, there would be a higher sound intensity on the second pass that would create an equal probability of response as the first pass, which is not present.

## Summary and conclusions

This paper reports on the behavioral responses of free ranging ungulate species (Oryx, Kudu, Springbok, Giraffe, Eland, Hartebeest, and Impala) housed in an animal reserve in Namibia to the presence of different UAV models. The study included 397 passes over 99 flights; however, the dataset only used 346 passes over 76 flights negating preliminary flights and only including properly performed flights at altitudes ranging from 15 m to 55 m in three categories of response level: No response, Alert, and Movement. The results suggest a stronger correlation between flight altitude and response across the different ungulates, and the evidence suggests rapid habituation to the UAVs.

The single herd and mixed herd observations conducted in the study provide evidence in support of the feasibility of aerial wildlife monitoring (AWM) by UAVs. The highest percentage of responses that did not invoke movement occurred at the highest altitudes. The results suggest that the longer the UAV was in the presence of the animals the less likely they were to negatively respond. Certain species were found to be more reactive than others, in addition to several displaying different response levels in single or mixed herd environments. Zebras were found to be less responsive in mixed herd environments while Oryx were present, as compared to when the Oryx were not; suggesting that some species may respond based on other species perception of threat or their relative fitness levels.

The UAVs also produced inconsistent response rates between movement and alert behavior. The reference vehicle, Phantom 3 was much more likely than the Mavic to induce an alert response, while both having similar probabilities of inducing a movement response. Furthermore, the Custom X8 showed significantly more alert and movement responses than the other UAVs. This shows there may be several aspects to the UAVs that affect the responses of the ungulates. For instance, the sound intensity may alert the species more often, but close proximity may induce a movement response. As seen with the reference vehicle, Phantom 3, there was a less than 7.5% chance of inducing a movement response at 55 meters. Additionally, after the average number of passes (4.5) while flying at 55 meters reduced the predicted probabilities of inducing an alert response from 46% to 10% and a movement response from 6% to 1% or an average reduction of approximately 80%. More generally, the data shows that when the UAV is flying above 50 meters and has a measured sound intensity below 50 dB, the likelihood of inducing a movement response on an ungulate species is below 6% regardless of the vehicle on the first pass over the animals.

The factors that contribute the most are not exactly known; however, sound profile (i.e., sound intensity, frequency), size, and style of flight (i.e., multirotor, fixed wing) likely play a roll. The study did not conclude any long-term effects of the habituation of the UAVs because it was very difficult to confirm that the same animals were being flown over on each subsequent day. This was due to the large size of the property and substantial number of animals. Further study of habituation to the presence of UAVs could further strengthen the results including studies monitoring the same animals over extended periods of time.

## Supporting information

S1 Questionnaire (DOCX)
